# A review of alternative methods to the use of animals in safety evaluation of cosmetics

**DOI:** 10.31744/einstein_journal/2022RB5578

**Published:** 2022-01-27

**Authors:** Barbarah Helena Nabarretti, Roberta Balansin Rigon, Jonny Burga-Sánchez, Gislaine Ricci Leonardi

**Affiliations:** 1 Faculdade de Ciências Médicas Universidade Estadual de Campinas Campinas SP Brazil Faculdade de Ciências Médicas, Universidade Estadual de Campinas, Campinas, SP, Brazil.; 2 Faculdade de Ciências Farmacêuticas Universidade Estadual de Campinas Campinas SP Brazil Faculdade de Ciências Farmacêuticas, Universidade Estadual de Campinas, Campinas, SP, Brazil.; 3 Faculdade de Odontologia de Piracicaba Universidade Estadual de Campinas Piracicaba SP Brazil Faculdade de Odontologia de Piracicaba, Universidade Estadual de Campinas, Piracicaba, SP, Brazil.

**Keywords:** Alternative methods, Toxicity, *In vitro* techniques, Cosmetics, Safety assessment, Animals

## Abstract

Alternative methods to the use of animals in research have been a global trend, mainly after the publication of the 3R’s principle (Replacement, Reduction, and Refinement), proposed by Russel and Burch. In the cosmetic sector, safety and efficacy assessments using animals have generated controversial debates. For this reason, *in vitro* research techniques are widely used to assess acute toxicity; corrosivity and irritation; skin sensitization; dermal and percutaneous absorption; repeated dose toxicity; reproductive toxicity; mutagenicity and genotoxicity; carcinogenicity; toxicokinetic studies; photo-induced toxicity; and human data. Although there are many methodologies described, validated, and widely used in the cosmetic area, the evaluation of the safety of cosmetic ingredients and products is still an expanding field. It needs global collaboration among regulatory agencies, universities, and industry, to meet several unmet needs in the fields of sensitization, carcinogenicity, systemic action, among other issues involving safety of users of cosmetic products. This review article will cover the currently most relevant *in vitro* models regarding irritation, corrosion, sensitization, mutagenicity, genotoxicity, and phototoxicity, to help to choose the most appropriate test for evaluating the safety and toxicity of cosmetic ingredients and products.

## INTRODUCTION

Organizations such as the Food and Drug Administration (FDA) in the United States, the Registration, Evaluation, Authorisation, and Restriction of Chemicals (REACH) in the European Union, and the National Health Surveillance Agency (Anvisa - *Agência Nacional de Vigilância Sanitária*) in Brazil, are responsible for the safety-related assessment guidelines for cosmetic products, which can be *in silico, in vitro*, and *in vivo.* At Anvisa, they are described in the Guide to Cosmetic Product Safety Evaluation (https://www.gov.br/anvisa/pt-br/centraisdeconteudo/publicacoes/cosmeticos/manuais-e-guias/guia-para-avaliacao-de-seguranca-de-produtos-cosmeticos.pdf/view).

With the introduction of the 3R’s principle (Replacement, Reduction, and Refinement) in 1959, by Russell et al., who published “The principles of humane experimental technique”, the search for the development of new methodologies aimed at reduction, refinement, and replacement of the use of animals in research has become a global trend.^(1)^

Currently, in Anvisa’s cosmetics area, no legislation obliges companies to present tests on animals. The Resolution of the Collegiate Board of Directors (RDC) 38/2001, (https://www.cosmeticsonline.com.br/ct/painel/fotos/assets/uploads/regulatorios/2e9f6-Rdc-38.pdf), which required submission of oral toxicity tests for registration of lipstick and lip gloss, rouge, and children’s blush, and Ordinance 1480, of December 31, 1990 (https://bvsms.saude.gov.br/bvs/saudelegis/gm/1990/prt1480_31_12_1990.html), which required the presentation of a primary cumulative skin irritation test, and dermal sensitization testing in animals, have now been replaced by new resolutions: respectively, RDC 15/2015 (http://bvsms.saude.gov.br/bvs/saudelegis/anvisa/2015/rdc0015_24_04_2015.pdf) and RDC 142/2017 (http://www.in.gov.br/materia/-/asset_publisher/Kujrw0TZC2Mb/content/id/20833442/do1-2017-03-20-resolucao-rdc-n-142-de-17-de-marco-de-2017-20833350) – the latter amended by RDC 178/2017 (http://sincamesp.com.br/wp-content/uploads/sites/74/2017/10/U_RS-MS-ANVISA-RDC-178_260917.pdf).

The RDC 35/2015 (https://bvsms.saude.gov.br/bvs/saudelegis/anvisa/2015/rdc0035_07_08_2015.pdf) describes alternative methods to the use of animals in research activities, accepted under Normative Resolution # 17, of July 3, 2014 (https://antigo.mctic.gov.br/mctic/export/sites/institucional/institucional/concea/arquivos/legislacao/resolucoes_normativas/Resolucao-Normativa-CONCEA-n-17-de-03.07.2014-D.O.U.-de-04.07.2014-Secao-I-Pag.-51.pdf). The National Council for the Control of Animal Experimentation (CONCEA - *Conselho Nacional de Controle de Experimentação animal*) has already published two Normative Resolutions recognizing 24 alternative methods: the Normative Resolution # 18, of September 24, 2014 (https://www.in.gov.br/web/dou/-/resolucao-normativa-concea-n-51-de-19-de-maio-de-2021-321534226) and the Normative Resolution # 31, of August 18, 2016 (https://antigo.mctic.gov.br/mctic/export/sites/institucional/institucional/concea/arquivos/legislacao/resolucoes_normativas/Resolucao-Normativa-CONCEA-n-31-de-18.08.2016-D.O.U.-de-19.08.2016-Secao-I-Pag.-04.pdf).

According to Anvisa, other methods may have regulatory acceptance, provided they have validated protocols, are internationally recognized by the Organisation for Economic Cooperation and Development (OECD) or a similar authority, and are part of a “phased alternative testing strategy”, also known as Integrated Approaches to Testing and Assessment.

There is also a guide from the Scientific Committee on Consumer Safety (SCCS) with requirements to be adopted for the evaluation of acute toxicity; corrosivity and irritation; skin sensitization; dermal and percutaneous absorption; repeated dose toxicity; reproductive toxicity; mutagenicity and genotoxicity; carcinogenicity; toxicokinetic studies; photo-induced toxicity; and human data.^(2)^

This review article discusses the most relevant *in vitro* models currently used for irritation, corrosion, sensitization, mutagenicity, genotoxicity, and phototoxicity, to help to select the most appropriate test for safety and toxicity evaluation of cosmetic ingredients and products.

## DESCRIPTION OF THE TECHNIQUES USED IN EVALUATION OF CYTOTOXICITY IN COSMETICS

Cytotoxicity testing is a widespread technique for evaluating the toxicity of cosmetic ingredients and products, which can be used in preclinical studies.

Most of the assays used to require an incubation time of the reagent with the cell population, and the viable cells must convert a substrate into a colored or fluorescent product that can be detected by equipment capable of absorbance or colorimetric reading.^(3)^

Among the most commonly used assays are MTT (3-(4,5-dimethylthiazol-2-yl)-2,5-diphenyltetrazolium bromide); sulforhodamine B (SRB); neutral red (NRU; 3-amino-7-dimethylamino-2-methyl phenazine); LIVE/DEAD™, and cell parameters obtained via flow cytometry.

Some interference may lead to different results in the cell viability tests, making it necessary to choose according to the application (Table 1).^(4-10)^

**Table 1:** Summary of *in vitro* techniques for assessing cell viability

Parameters	MTT assays	SRB assay	NRU assay	LIVE/DEAD™ assay	Flow cytometry
Mechanism of action	Reduction of formazan via NADH electron transfer to form MTT	Binds to protein components of cells that have been fixed on culture plates by TCA	Viable cells bind to the dye and attach themselves by hydrophobic electrostatic bonds to anionic sites in the lysosomal matrix	Calcein AM is broken down by non-specific esterases, resulting in a green fluorescent compound	Measures optic and fluorescent characteristics of a single cell or other particles in a fluid stream as they pass through a light source
Interferents and limitations	Glycolysis inhibitors, antioxidants, polyphenols, nanovectorized titanium dioxide, vitamins, dyes, magnesium, copper, and liposomes^(4)^	Rarely presents interferences, but evaluates the entire protein content, requiring removal of dead cells from the plate to determine cell viability^(5)^	pH-dependent absorption of the dye in the viable cell matrix. Not suitable for volatile, water unstable, and low solubility substances^(6)^	Nanoparticles favor PI entry and increase false-positive results; higher cost than other techniques^(7)^	High cost of instruments and markers, specialists for execution is needed, limited in the analysis of tissue architecture and intercellular interactions^(8)^
Advantages	Fast, easy-to-handle, low-cost model, with wide use and reproducible results	Few described interferences. Best linearity, high sensitivity, stability over time, and low cost. Can be used in formulations with sunscreens and antioxidants^(9)^	In *vitro/in vivo* correlation in the 95% range; low cost, no unstable reagents^(6)^	High sensitivity, fast and simple handling	A rapid and reliable method capable of promoting a quantitative evaluation of viable cells in suspension and multiple cellular processes, and assessing the type of cell death type^(10)^
Incubation period	1-4 hours	2-3 hours	3.5 hours	1 hour	Depends on the antibody -- no more than 15 to 30 minutes

MTT: 3-(4,5-dimethylthiazol-2-yl)-2,5-diphenyltetrazolium bromide; SRB: sulforhodamine B; NRU: neutral red; NADH: nicotinamide adenine dinucleotide adenine; TCA: trichloroacetic acid; AM: calcein acetoxymethyl ester; PI: propidium iodide.

## DESCRIPTION OF THE TECHNIQUE USED IN THE EVALUATION OF PHOTOTOXICITY IN COSMETICS

Phototoxicity is an acute reaction triggered after exposure to solar, ultraviolet, or visible radiation, due to the application of a chemical product topically or systemically.^(11)^

The *in vitro* 3T3 NRU-ultraviolet assay (95% correlation with *in vivo* assay) uses standard fibroblast cells obtained from Swiss mouse embryonic tissue cells (3T3), and neutral red dye to compare the 50% mean inhibitory concentration (IC50), with and without exposure to solar radiation.^(11)^

## DESCRIPTION OF TECHNIQUES USED IN EVALUATION OF MUTAGENICITY AND GENOTOXICITY IN COSMETICS

Ames’ test is recommended by the OECD # 471 guide and by the safety guidelines of Anvisa (https://www.oecd.org/chemicalsafety/risk-assessment/1948418.pdf) for the assessment of mutagenicity. In the test, *Salmonella typhimurium* with pre-existing mutations does not synthesize histidine (does not form colonies) and is treated with mutagenic chemical ingredients making mutations occur, which restore the ability of bacteria to deform colonies.^(12)^

The micronucleus, on the other hand, is formed from DNA fragmentations of bone marrow erythrocytes, after exposure to genotoxic substances, shown after cell division. Changes in pH or osmolarity and interactions of the substance with the cell culture medium can generate false-positive results.^(13)^

Micronucleus testing can also be performed on erythrocytes from fertilized chicken eggs (HET-MN, hen’s egg test for micronucleus induction), in which the chorioallantoic membrane (CAM) is exposed to a solution of the test substance. This model is the most comparable to the *in vivo* system due to the metabolic capacity of the egg, although it has not yet been validated by competent bodies, such as the European Centre for the Validation of Alternative Methods (ECVAM).^(14)^

## DESCRIPTION OF TECHNIQUES USED IN COSMETIC SKIN AND EYE IRRITATION ASSESSMENT

Reconstructed human epidermis is defined as a three-dimensional reconstruction of primary keratinocytes extracted from human skin, amplified on an inert polycarbonate filter or collagen matrix, and exposed to an air-liquid interface, causing differentiation from the stratified epidermis.^(15)^

EpiSkin™, EpiDerm™, and SkinEthic™ are OECD validated models that have reasonable similarities to natural human skin.^(16-18)^All models present with stratum corneum, spinosum, granulosum, and basale, as well as natural human skin, with a relatively thicker stratum corneum in the *in vitro* models (12µm to 37µm) compared to natural human skin (10µm to 12µm), because of the absence of cutaneous desquamation in the models.

Regarding lipid composition, there are some discrepancies compared to *in vitro* models. EpiDerm™ is most similar to natural human skin in terms of the amount of phospholipids, di-/triglycerides, and ceramides-4. SkinEthic™ is similar in amount to free fatty acids, ceramides-2a, and ceramides-3, and EpiSkin™ to cholesterol and ceramides-1, relative to natural human skin.^(16-18)^

Natural human skin expresses some enzymes and proteins, such as keratin-1/10, loricrin, involucrin, and transglutaminase, which are also present in the validated models.^(16-18)^

Keratin-6 and skin-derived antileukoproteinase (SKALP) are absent in natural human skin, but are present in the three models described.^(16-18)^

Although the models have only the skin epidermis in their composition, recent research using models containing both epidermis and dermis (reconstructed human skin) seeks the understanding of physiological changes in aging skin, as well as with skin pathologies for the validation of reconstructed human skin models that can be representative of these conditions, contributing to improving the pharmacological treatment of skin diseases.^(19)^

**Figure 1:**
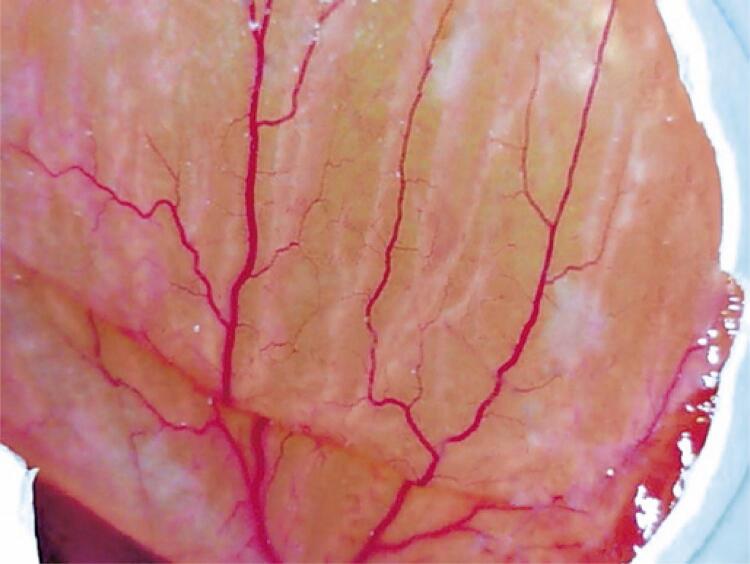
Chorioallantoid membrane with exposed blood vessels

Source: Image captured using a Dino-lite AM211 microscope and processed using ImageJ 1.52a software).

For eye irritation and toxicity, the hen’s egg test on chorioallantoic membrane (HET-CAM) has been used, which is performed by exposing the CAM of the fertilized chicken egg, as shown in figure 1. The compound to be evaluated is then applied to this membrane and, for 300 seconds, the antiangiogenic potential of the test substance is evaluated through a mathematical equation, whose variables correlate the moments in which the first signs of hemorrhage, vascular lysis, and coagulation occur. Irritation scores ranging from non-irritating to severely irritating have already been described.^(20)^

## DESCRIPTION OF TECHNIQUES USED IN THE EVALUATION OF SKIN SENSITIZATION IN COSMETICS

Some *in vitro* methods can be used to evaluate the sensitizing potential of test substances. These include the 442C^(21)^ and 442E^(22)^ tests described in the OECD guideline, and the assay KeratinoSens™.^(23)^

The molecular signaling of the onset of sensitization adverse outcome pathway, described in test 442C, is characterized by the covalent binding of electrolyte substances to nucleophilic centers of skin proteins, causing direct peptide reactivity assay (DPRA) skin sensitization, which is measured by high-performance liquid chromatography (HPLC). The percentage peptide depletion values are calculated for each sample, allowing a reactivity classification to be assigned.^(21,24)^

The human cell activation test (h-CLAT), test # 442E,^(22)^ evaluates changes in the expression of cell surface markers, such as CD86 and CD54, associated with the activation process of monocytes and dendritic cells in the THP-1 cell line, following exposure to potentially sensitizing substances.

The KeratinoSens™ assay uses immortalized human keratinocyte (HaCaT) lineage transfected with a selected plasmid, with the purpose of quantifying the gene induction of luciferase as a marker of Keap1-Nrf2-ARE pathway activation (nuclear factor erythroid2-related to factor 2-Kelch-like ECH protein 1; antioxidant response element). Such an *in vitro* method has been validated to assess the sensitization potential of chemical substances.^(23)^

## DISCUSSION

There is a wide variety of *in vitro* assays for the safety evaluation of cosmetic ingredients and products, aiming to meet the 3R’s principles of replacing animal use. Although several advances have been made in this area, much research is still needed to reduce or overcome the limitations inherent to *in vitro* assays.^(17,18)^

Regarding assays that evaluate cell viability, a variety of methodologies are available, but the assays measure only one cell parameter, making them restrictive and prone to failure. For example, the MTT assay relies on the quantification of the conversion of this substrate into formazan crystals by live cells, which determines mitochondrial activity after the treatment performed.^(25)^ In the LIVE/DEAD™ test, live cells convert non-fluorescent permeable calcein (calcein-AM) into green fluorescent impermeable calcein, which is distributed uniformly throughout the cytoplasm of the cell by the activity of esterases. Dead or membrane-damaged cells are labeled with ethidium homodimer-1 (EthD-1) by binding to nucleic acids, emitting red fluorescence, and this marker is excluded by the membranes of intact cells. In this way, it is possible to make both a qualitative and quantitative analysis of the cytotoxicity of a given substance.^(26)^ In the cell proliferation test using SRB as a reagent, SRB binds to the basic amino acids of proteins in viable cells at the time of fixation, and the greater the amount of SRB bound in the cells, the lower the cytotoxic activity of the test sample.^(27)^ Therefore, the selection of cell viability assays is a complex process that takes into account different factors, such as the nature and duration of the test, the required detection mechanism, the chemical structure of the compound to be evaluated, and the assay limitations themselves, which may generate similar quantitative cytotoxicity results.^(28)^ The discrepancies between the methods used by research groups reinforce the growing need for the development of a standardized guide that ensures rapid and reliable results in cell viability assays.

Commercial reconstructed human epidermis models have reasonable similarities to natural human skin and are important tools in ensuring the safety of a pharmaceutical and/or cosmetic substance. However, human skin models so far do not contain hair follicles, sebaceous glands, nerves, circulatory and lymphatic systems, which make similarity to *in vivo* studies difficult.

Although *in vitro* techniques are not yet able to fully replace *in vivo* techniques, for some assays, however, such as the Draize test, there is already a correlation with *in vitro* methods, such as the HET-CAM assay. Although not validated for regulatory purposes, it is accepted by Anvisa in screening assays.

Regarding the assays briefly summarized in this article and the others that exist, one should always be aware of their advantages, disadvantages, and limitations. An example is the technique described by the OECD test # 442E, which, although it has excellent precision in distinguishing among Category 1 (GHS) substances, and high specificity when compared to the *in vivo* local lymph node assay, is not able to identify substances that have mild to moderate irritancy potential.

Frequently, technical limitations regarding the nature of the test substance, such as solubility in the culture medium, may also occur. Moreover, in the *in vitro* models, the interaction between tissue and organ cannot be evaluated, as well as the systemic and chronic effects, and the pharmacokinetics cannot be established; hence, the compound needs to be evaluated under different conditions.^(29)^

In this way, the OECD guidelines themselves (https://www.oecd-ilibrary.org/environment/oecd-guidelines-for-the-testing-of-chemicals-section-4-health-effects_20745788) recommend the supplementation between techniques, to guarantee results that are more faithful to reality, avoiding underestimating, or overestimating the toxicity of a certain compound, and so that the mechanisms involved are better understood.

Thus, research related to the implementation of *in vitro* methodologies for safety assessment of cosmetic ingredients and products is still an expanding field in need of global collaboration between regulators, universities, and industry to address several unmet needs in the fields of sensitization, carcinogenicity, and systemic action - among other issues involving cosmetic user safety.
